# Supplementary material for: High Seroprevalence of Anti-SARS-CoV-2 Antibodies Among Ethiopian Healthcare Workers
Source: Res Sq. 2021 Jul 19:rs.3.rs-676935. Preprint. [Version 1] doi: 10.21203/rs.3.rs-676935/v1 (PMC8312903; doi:10.21203/rs.3.rs-676935/v1)
Supplement: Supplement 1 [file 92610fe5053ee1218a9d3cbf.docx]

**Table S1.** The Specificity of RBD IgG ELISA among pre-COVID-19 pandemic sera (n=365)

| Negative Samples | Total number tested | Negative | Specificity (%) | 95% CI |
| --- | --- | --- | --- | --- |
| Pre-COVID-19 pandemic sera | 364 | 350 | 97.7 | 95.6-99.0 |
| NIBS UK adults pooled plasma | 1 | 1 |  |  |
| Total | 365 | 351 | 97.7 | 95.6-99.0 |

**Table S2.** Sensitivity of RBD IgG ELISA among cohort COVID-19 patients (n=405) confirmed by RT-PCR.

| Sample collection | Total N Tested | Positive | Sensitivity (%) | 95% CI |
| --- | --- | --- | --- | --- |
| 1-7dps | 336 | 226 | 67.3 | 62.0-72.3 |
| 8-14dps | 52 | 39 | 75.0 | 61.0-86.0 |
| 15-21dps | 13 | 13 | 100.0 | 75.29-100.0 |
| NIBSC UK COVID-19 convalescent plasma | 4 | 4 | 100.0 | - |
| Total Convalescent samples s | 17 | 17 | 100.0 | 84.2-100 |

**Table S3. Percentage of positive specimens (n=40) from patients who tested positive for SARS-CoV-2 by DAAn RT–PCR**

| Assay | Sample collection | IgM | | IgG | | Pan igs | |
| --- | --- | --- | --- | --- | --- | --- | --- |
|  |  | **%** | **95% CI** | **%** | **95% CI** | **%** | **95%** |
| In-house ELISA^a^ |  |  |  |  |  |  |  |
|  | 1-7 dps^b^ | **na** | **na** | **73.3** | 44.9- 92.2 | na | na |
|  | 8-14 dps |  |  | **86.7** | 59.5- 98.3 |  |  |
|  | 14-21 dps | **na** | **na** | **100.0** | 69.1-100.0 | na | na |
| Wantai ELISA |  |  |  |  |  |  |  |
|  | 1-7 dps | **na** | **na** | **na** | **na** | **66.7** | 38.4-88.2 |
|  | 8-14 dps |  |  |  |  | **86.7** | 59.5- 98.3 |
|  | 14-21 dps | **na** | **na** | **na** | **na** | **100.0** | 69.2-100.0 |
| REALY LFA^c^ |  |  |  |  |  |  |  |
|  | 1-7 dps | **80.0** | 51.9- 95.7 | **80.0** | 51.9- 95.7 | na | na |
|  | 8-14 dps | **86.7** | 59.5- 98.3 | **80.0** | 51.9- 95.7 |  |  |
|  | 15-28 dps | **90.0** | 55.5-99.0. | **90.0** | 55.5-99.8 | na | na |

^a^dps=days post symptoms; ^a^ ELISA=Enzyme-Linked Immunosorbent Assay; ^b^ na=not applicable;^bc^ LFA=Lateral Flow Assay

**Table S4. Specificity of RBD IgG ELISA in pre-covid plasma/serum specimens (n=40) collected before COVID-19 pandemic**

| Assay | Specificity (%) | 95% CI |
| --- | --- | --- |
| In-house ELISA | **97.5** | 86.8- 99.9 |
| Wantai ELISA | **100.0** | 89.72-100.0 |
| Realty LFA | **92.5** | 79.6-98.4 |

**Supplementary Method**

Microtiter plates were coated with purified recombinant proteins of receptor binding domain of the spike protein of SARS-CoV-2 (100 μl/well) diluted in phosphate buffered saline, PBS (pH 7.4) at concentration 1 μg/mL and incubated overnight at 4 °C. Next day, excess unbound antigen was removed and thereafter microtiter plates were blocked with 300 μl/well of 4% skimmed milk with PBS plus 0.1% Teween-20 (w/v) for 2 hours at room temperature (RT). Following blocking step, microtiter plates washed 3X with PBS plus 0.05% Tween-20 (PBST) and thereafter 100 μl/ml of serum sample diluted at 1:200 in blocking buffer was added and incubated at RT for 60 min. Following incubation and 5X washes with PBST, 100 μl/well of horseradish peroxidase-conjugated anti-human immunoglublunin G (IgG) (Invitrogen, USA) diluted at 1:5000 in blocking buffer was added and incubated for 1 h at RT. After 5X washes, the reaction was visualized by adding 75 μl/well 3,3_,5,5_-Tetramethylbenzidine (TMB) liquid substrate (BioRad, USA) and incubating at RT in the dark for 10-15 min. The reaction was then stopped with 75 μl/well TMB stop solution. The optical density (OD) was measured at 450 nm filter on ELISA LT-45000 microplate reader. Each sample was tested in duplicate.
